# Use it or lose it: establishment and persistence of T cell memory

**DOI:** 10.3389/fimmu.2012.00357

**Published:** 2012-11-27

**Authors:** Katherine Kedzierska, Sophie A. Valkenburg, Peter C. Doherty, Miles P. Davenport, Vanessa Venturi

**Affiliations:** ^1^Department of Microbiology and Immunology, University of MelbourneMelbourne, VIC, Australia; ^2^Department of Immunology, St Jude Children’s Research HospitalMemphis, TN, USA; ^3^Complex Systems in Biology Group, Centre for Vascular Research, University of New South WalesKensington, NSW, Australia; ^4^Computational Biology Group, Centre for Vascular Research, University of New South WalesKensington, NSW, Australia

**Keywords:** T cell memory, CD8 T cells, aged, Influenza A virus, CD4^+^ T lymphocytes

## Abstract

Pre-existing T cell memory provides substantial protection against viral, bacterial, and parasitic infections. The generation of protective T cell memory constitutes a primary goal for cell-mediated vaccines, thus understanding the mechanistic basis of memory development and maintenance are of major importance. The widely accepted idea that T cell memory pools are directly descended from the effector populations has been challenged by recent reports that provide evidence for the early establishment of T cell memory and suggest that the putative memory precursor T cells do not undergo full expansion to effector status. Moreover, it appears that once the memory T cells are established early in life, they can persist for the lifetime of an individual. This is in contrast to the reported waning of naïve T cell immunity with age. Thus, in the elderly, immune memory that was induced at an early age may be more robust than recently induced memory, despite the necessity for long persistence. The present review discusses the mechanisms underlying the early establishment of immunological memory and the subsequent persistence of memory T cell pools in animal models and humans.

## INTRODUCTION

Memory T cells provide protection against re-infection with the same pathogen enabling a more rapid recovery of the host and a milder clinical outcome (Powell et al., 2007). For example, survival from the H2N2 influenza pandemic of 1957 was attributed to pre-existing T cell immunity in the adult population (Epstein, 2006). Furthermore, high levels of pre-existing CD4^+^ T cell memory correlate with reduced viral loads and clinical scores in a human influenza challenge study, highlighting the importance of T cell memory in viral infection (Wilkinson et al., 2012). Once established, memory T cells can reside in tissues or recirculate between the blood, organs, and lymph nodes. These memory T cells appear to be quite distinct from the recently described tissue resident memory cells (Trm; Gebhardt et al., 2009) displaying a unique set of molecular markers and optimal functions within the tissues (Mackay et al., 2012; Wakim et al., 2012).

For a long time, the traditional view of T cell memory formation involved the expansion of pathogen-specific effector T cells in number in response to infection followed by an extensive contraction in cell numbers (~90–95%), leaving a residual stable memory T cell pool, known as the linear model of memory differentiation. It has been known for decades that CD8^+^ memory T cells sort into CD62L^hi^ and CD62L^lo^ sets (Tripp et al., 1995), with the former having the capacity to recirculate from blood to lymph nodes via the high endothelial venules, while the latter move readily through other tissue compartments and can return to the lymph node via the afferent lymphatics. Thirteen years ago, a seminal paper by Sallusto et al. (1999) coined the terms “central” (CD62L^hi^) and “effector” (CD62L^lo^) to describe these memory T cell subsets. These findings challenged the traditional view of memory formation, suggesting a more complex process, and raised important questions about the generation of T cell memory and which populations actually persist for the life-time of an individual. In fact, the key question has long been whether persistent T cells (CD62L^hi^ or CD62L^lo^) are survivors from the clonally expanded effector cytotoxic T lymphocyte (CTL) pool or are part of a separate lineage.

More than a decade of research and published studies provide evidence of the early establishment of memory, which can be greatly affected by the inflammatory stimuli and transcription signatures at the acute phase of the infection. Both animal and human studies also show that T cell memory is long-lived and can be detected for up to 75 years in humans (Hammarlund et al., 2003) and for the life-time of a laboratory mouse (Valkenburg et al., 2012). A growing area of research focused on the impact of age on the immune system suggests that age-related changes have potential implications for the generation and maintenance of T cell memory late in life. Interestingly, memory T cell populations directed at acute and long-term persistent infections exhibit different functions and characteristics, as the latter display signs of exhaustion due to the persistence of the antigen. In addition, studies suggest that, in the context of acute infections, the protective capacity of memory CD8^+^ T cells is pathogen-specific and can be substantially impacted by repeated antigenic stimulation (Nolz and Harty, 2011). In this review, we discuss both the establishment and persistence of primary T cell memory, with a particular focus on CD8^+^ T cell responses directed at acute readily resolved infections such as respiratory viruses.

## ESTABLISHMENT OF T CELL MEMORY

The relatively recent development of the tetramer-magnetic enrichment approach (Moon et al., 2007) has substantially shaped our understanding of the recruitment, expansion, and persistence of endogenous T cell numbers for the naïve, effector, and memory T cell populations. The numbers of naïve T cells per mouse available to respond to a specific antigenic epitope range from tens (e.g., ≈30 naïve precursors for the immunodominant influenza-specific D^b^NP_366_ epitope; La Gruta et al., 2010) to hundreds (e.g., ≈600 for the murine cytomegalovirus D^b^M45 epitope; Obar and Lefrancois, 2010). After antigenic stimulation, the naïve precursors increase in prevalence by more than 10,000 times (Croom et al., 2011), whilst substantially fewer cells establish stable memory pools. These memory pools increase the numbers of antigen-specific T cells by up to 200–1000 times (Belz et al., 2000; Hogan et al., 2001; Marshall et al., 2001; Croom et al., 2011) compared to the initial naïve precursor pool (La Gruta et al., 2010). Such effects are readily observed in mouse model systems following prime/challenge with influenza A viruses that differ for their surface H and N proteins but share internal components (Kedzierska et al., 2008). It has been reported that the size of antigen-specific memory pools strongly correlates with the level of immune protection (Badovinac et al., 2003; Schmidt et al., 2008). As the establishment of immunological memory is critical for a rational design of any cell-mediated vaccine, understanding the precise mechanisms of memory formation is essential.

### EARLY ESTABLISHMENT OF T CELL MEMORY

Recent studies provide important insights into the development of memory T cells. Experiments using *in vitro* stimulated T cells (Kaech and Ahmed, 2001), antibiotic treatment prior to *Listeria monocytogenes* or influenza co-infection (Badovinac et al., 2004), dendritic-cell vaccination (Badovinac et al., 2005), clonal dissection of influenza-specific CD8^+^ T cells at different stages of infection (Kedzierska et al., 2006, 2008) or transfer studies (Kedzierska et al., 2007) suggest that the full expansion to effector status is not a pre-requisite for the generation of long-term memory T cells.

As mentioned previously, the discovery that memory T cell populations are comprised of distinct T cell subsets (Sallusto et al., 1999) has also had a substantial impact on our understanding of T cell memory. Based on the expression of lymph-node homing markers (CD62L and CCR7), memory T cells have been classified as central memory T cells (T_CM_; CD62L^hi^, CCR7^hi^), circulating between lymphoid organs, and effector memory (T_EM_, CD62L^lo^, CCR7^lo^), found principally in the blood, spleen, and peripheral organs (Sallusto et al., 1999; Masopust et al., 2001; Reinhardt et al., 2001). These T_CM_ and T_EM_ memory populations are also considered to differ at the functional level. While human T_EM_ cells display immediate cytotoxic activity *ex vivo*, T_CM_ populations acquire effector function after short-term *in vitro* stimulation (Sallusto et al., 1999; Masopust et al., 2001). Although both subsets can produce anti-viral cytokines IFN-γ and TNF-α, IL-2 production is largely limited to CD62L^hi^ memory cells (Wherry et al., 2003). Significant insights into T cell formation and maintenance have been gained since studies have focused on classifying T_EM_ and T_CM_ cells.

**FIGURE 1:**
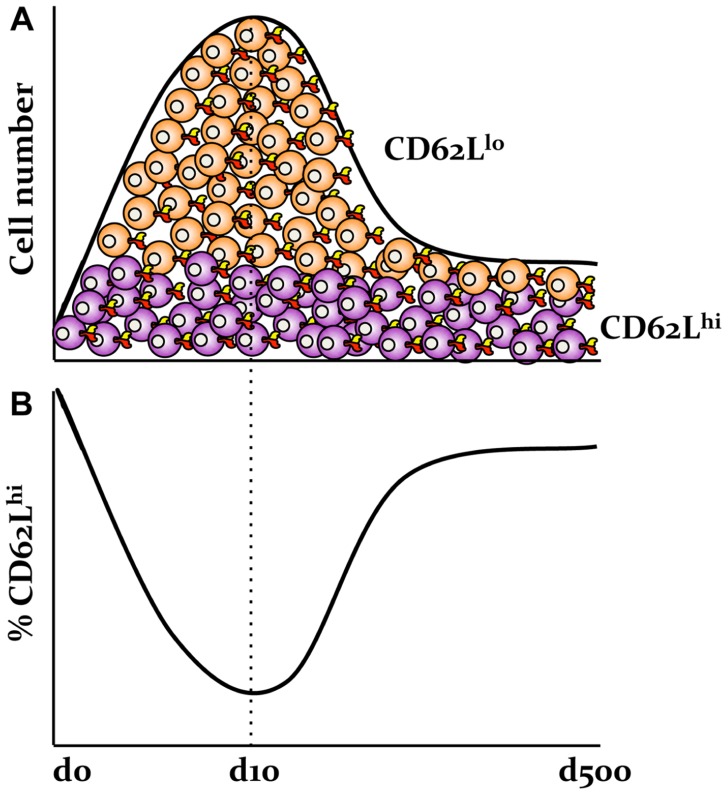
**Preferential survival of antigen-specific memory CD62L^hi^ CD8^+^ T cells following influenza virus infection**. **(A)** The numbers of influenza-specific CD62L^hi^CD8^+^ T cells, but not CD62L^lo^ CD8^+^ T cell sets, are stable following the primary infection. **(B)** This is in contrast to the relative frequencies of CD62L^lo^ and CD62L^hi^ effector and memory T cell populations as based on our published data in Kedzierska et al. (2006).

A first important observation from studies of the T_EM_ and T_CM_ subsets is that the proportions of T_EM_ and T_CM_ populations change throughout the course of infection. In contrast to naïve CD8^+^ T cells expressing the CD62L^hi^ CD44^lo^ phenotype, the majority of CD8^+^ T cells present at the peak of infection display the surface markers CD62L^lo^ CD44^hi^, although some (around 10%; Kedzierska et al., 2006) remain of the CD62L^hi^ CD44^hi^ phenotype (**Figure 1**). As the virus is cleared and memory is established, the proportion of cells with the CD62L^hi^ phenotype progressively increases. For example, around 50% of the influenza-specific immunodominant D^b^NP_366_^+^CD8^+^ and D^b^PA_224_^+^CD8^+^ T cell memory populations express CD62L^hi^ at d60 after infection, compared with 90% being CD62L^hi^ by d500 (Kedzierska et al., 2006). Interestingly, the numbers of CD62L^hi^ CD8^+^ T cells remain relatively stable throughout the infection, while the total numbers of CD62L^lo^ CD8^+^ T cells rapidly decreases during the memory phase (Schlub et al., 2010; **Figure 1A**). This suggests that, rather than there being a regression from CD62L^lo^ to CD62L^hi^, there is preferential survival of antigen-specific memory CD62L^hi^ CD8^+^ T cells (T_CM_) set. This can be also translated into the relative contributions of CD62L^lo^ and CD62L^hi^ CD8^+^ T cell memory populations to the secondary response to Sendai virus (Roberts and Woodland, 2004; Roberts et al., 2005). Although the recall responses by the CD62L^lo^ CD8^+^ T cell memory population dominate at the early memory phase of infection (d30), the central memory CD62L^hi^ CD8^+^ T cells from later (1 year) memory time-points make a markedly greater contribution to recall responses (Roberts et al., 2005).

**FIGURE 2:**
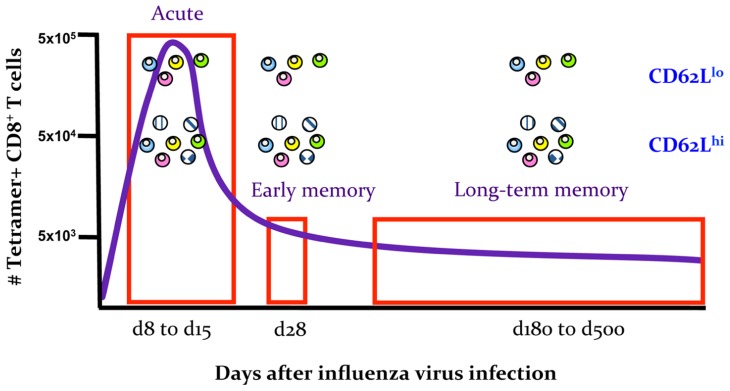
**Early establishment of a stable TCR repertoire composition for memory T_EM_ CD62L^lo^ and T_CM_ CD62L^hi^ sets**. Schematic summarizing the results of TCR repertoire analysis of the T_EM_ CD62L^lo^ and T_CM_ CD62L^hi^ subsets for influenza-specific D^b^NP_366_^+^ and D^b^PA_224_^+^CD8^+^ T cells at the acute phase of infection (d8–d15), early memory (d28) and late memory (d180–d500). Data are based on *n* = 3018 sequences published in Kedzierska et al. (2006).

**FIGURE 3:**
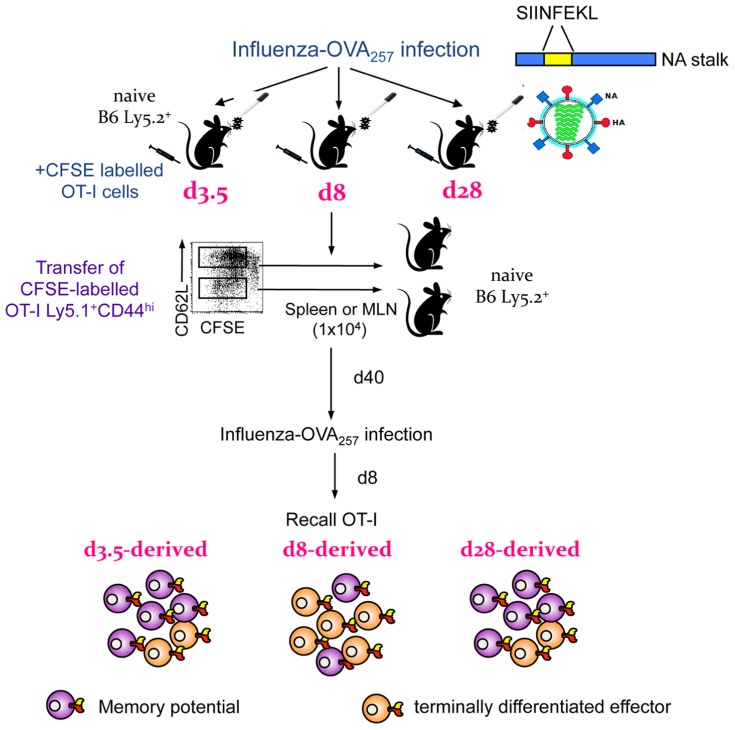
**Early establishment and recall of memory precursors in an OT-I transgenic system**. Diagrammatic representation of findings published in Kedzierska et al. (2007). OT-I cells were labeled with CFSE, transferred into congenically different B6 mice and infected with an influenza virus expressing the SIINFEKL peptide in the viral NA. On d3.5, d8, or d28 after infection, OT-I CD44^hi^ cells, which have divided, were transferred into naïve B6 mice. After the following 40 days, mice were infected with influenza expressing SIINFEKL to recall any surviving memory cells. OT-I cells from d3.5 after influenza infection could survive and be recalled, findings which reflected the d28 (memory) time-point. The memory potential of d8 effectors was greatly diminished, most likely due to a high proportion of terminally differentiated effectors.

Secondly, important insights into the early establishment of T cell memory have been made possible by the differential expression of CD62L^lo^ and CD62L^hi^ throughout the memory phase, which allows the tracking of different subsets starting from the early days after infection. Experiments utilizing transgenic mice suggest that it is possible to transition from the “more activated” CD62L^lo^ to the “less activated” CD62L^hi^ phenotype, indicating that being CD62L^lo^ is not a marker of terminal differentiation and supporting the possibility that there can be a linear model of T_EM_ to T_CM_ memory generation (Wherry et al., 2002). The results of clonotypic analysis of T cell receptor (TCR) signatures in mice (Bouneaud et al., 2005; Kedzierska et al., 2006) and humans (Baron et al., 2003) further expand this model. The indications are that although common TCRs are found in both T_CM_ and T_EM_ populations, the T_CM_ set contains additional TCR clonotypes not found within the T_EM_ set and that TCR diversity within both the T_CM_ and T_EM_ subsets appears to be stable. In mice, such divergent (and consistent) TCR repertoire characteristics are apparent for the T_CM_ and T_EM_ sets from the early acute phase of infection (d8) through to the long-term memory (>d500; **Figure 2**). This indeed suggests the early establishment of clonotypically stable T cell memory pools. In a follow-up study, we found that influenza-specific CD8^+^ T cells isolated on d3.5 after influenza infection, especially cells isolated from the draining lymph nodes, could survive following the transfer into naïve mice and be recalled after a secondary challenge (Kedzierska et al., 2007). This suggests that stable CD8^+^ T cell memory is established early in the antigen-driven phase of influenza virus infection. The survival of memory CD8^+^ T cells isolated early after infection on d3.5 highly resembled the survival of CD8^+^ T cells isolated during the memory phase (d28; **Figure 3**). Conversely, when CD8^+^ T cells isolated at d8 (i.e., at the peak of the acute phase) were transferred into a naïve animal, the survival potential was lower than those of cells isolated early after the infection. This most likely reflects the high proportion of terminally differentiated short-lived effectors at the peak of the response to influenza viruses (**Figure 3**). Overall, these findings support the idea that antigen-specific CD8^+^ T cells that have not achieved full effector potential are more likely to be part of the memory pool. For example, CD8^+^ T cells lacking the cytotoxic capacity thus protected from cell damage during target cell killing. In addition, early d3.5 CD8^+^ T cells lacking CD25 (the IL-2Rα subunit) displayed enhanced survival into memory (Kedzierska et al., 2007). This was further confirmed, more recently (Belz and Masson, 2010; Kalia et al., 2010; Pipkin et al., 2010) in studies which showed lower CD25 expression, and thus weaker IL-2 signaling, leads to preferential generation of T cells with a memory phenotype capable of long-term survival.

Expression of the IL-7R α-chain (CD127) on CD8^+^ T cells during the acute phase of the response is also essential, although not sufficient on its own, for identifying the memory precursors at the acute time of infection (Kaech et al., 2003; Huster et al., 2004; Hand et al., 2007; Croom et al., 2011). Evidence for this comes from studies showing that, IL-7Rα-expressing cells at the peak of LCMV infection survive preferentially to give long-lived memory and show increased expression of anti-apoptotic molecules (such as bcl-2; Kaech et al., 2003). Further, FACS separation of LCMV-specific effector CD8^+^ T cells on the basis of IL-7Rα and KLRG1 expression led to characterization of the IL-7Rα^hi^ KLRG1^lo^ memory precursor effector cells (MPEC), as distinct fromIL-7Rα^lo^ KLRG1^hi^ short-lived effector cells (SLEC) following LCMV infection (Joshi et al., 2007). Acquisition of this memory IL-7Rα^hi^ KLRG1^lo^ phenotype in models of rapid, systemic infection (LCMV and *L. monocytogenes*) depends on the spectrum of inflammatory cytokines present during the acute phase of the disease process (Joshi et al., 2007; Pham et al., 2009).

In influenza infection, a substantial number of antigen-specific D^b^NP_366_^+^CD8^+^ and D^b^PA_224_^+^CD8^+^ T cells express IL-7Rα at the peak of the acute primary response (70.2 ± 1.5 and 37.5 ± 2.4%, respectively), which does not translate into the relative numbers of T cells in the stable memory pools (Croom et al., 2011). Thus, at least for influenza in a non-TCR-transgenic B6 mouse system, the majority of the antigen-specific IL-7Rα^+^ CD8^+^ T cells do not survive into the memory phase in a non-TCR-transgenic B6 mouse system. Further subsetting of influenza-specific CD8^+^ T cells into the SLEC and MPEC sets showed that, although the IL-7Rα^hi^ KLRG1^lo^ CD8^+^ population recovered from the spleen and the infected respiratory tract showed evidence of enhanced survival, the number of cells of this phenotype at peak does not define memory numbers. Our data suggested that the most stable MPEC IL-7Rα^hi^ KLRG1^lo^ numbers are observed in the draining mediastinal LN (MLN, draining the lungs), providing further evidence that, following this localized respiratory infection, the draining MLN offers the optimal anatomical niche for memory establishment and maintenance after influenza infection. Furthermore, the IL-7Rα^hi^ KLRG1^lo^ phenotype (but not the ultimate size of the memory pool) can be altered by varying the antigen dose, antigen presentation, extent of T cell division, and CD8^+^ T cell precursor numbers (Croom et al., 2011). This indicates that cell surface molecule expression on CD8^+^ T cells predominantly reflects early antigenic experience and to a lesser extent marks the capacity to generate CD8^+^ T cell memory. And obviously, the potential of the memory cells to survive can be coupled to its replicative capacity (Hikono et al., 2007).

In addition to the important determinants of T cell memory generation discussed above, sufficient CD4^+^ T cell help is also required for the establishment of stable memory (but not effector) influenza-specific CD8^+^ T cell populations. This was established by the diminished magnitude of memory and recall CD8^+^ T cell responses in MHC class II^-^ (IA^b^^-^^/^^-^) mice primed and then challenged with the influenza viruses (Topham et al., 1996; Doherty and Christensen, 2000; Riberdy et al., 2000; Belz et al., 2002), and mice treated with monoclonal antibody against CD4^+^ T cells (McKinstry et al., 2012; Swain et al., 2012). Unhelped CD8^+^ T cells have reduced survival to memory and reduced expansion during recall. The mechanism proposed involves help from CD4^+^ T cells resulting in down-regulation of TNF-α related apoptosis inducing ligand (TRAIL) on CD8^+^ T cells (Janssen et al., 2005; Badovinac et al., 2006).

### THE ROLE OF INFLAMMATION AND TRANSCRIPTION FACTORS IN THE GENERATION OF STABLE MEMORY CD8^+^ T CELL POOLS

Infection-induced inflammation can affect T cell immunity at any stage. Evidence suggests that the fate of memory precursors can be determined by the nature of inflammatory stimuli during the antigen driven phase. Elegant studies using antibiotic treatment prior to *L. monocytogenes* infection showed that reduced inflammation (including diminished IFN-γ production) markedly decreased T cell contraction (Badovinac et al., 2004). Conversely, increasing inflammation with CpG treatment restored this effector T cell editing process (Badovinac et al., 2004). These findings suggest that T cell memory pools can be generated independent of whether there is massive contraction in numbers. Furthermore, IL-12 has been shown to affect the progression to MPEC status via decreased T-bet expression (Joshi et al., 2007). While low inflammation (low T-bet expression) promotes MPEC formation, high inflammation (T-bet expression) leads to numerically enhanced SLEC population. Thus, it appears that the inflammatory milieu, and particularly IFN-γ and IL-12, impact the proportion of cells expressing effector and memory-like markers at peak, and therefore the establishment of T cell memory pools. However, the exact mechanisms by which inflammation affects memory formation are yet to be elucidated.

Studies using peptide-pulsed dendritic cells revealed that antigenic stimulation of T cells without high-level inflammation typically present in infection, led to the earlier establishment of T cell memory, both phenotypically and functionally (Badovinac et al., 2005). Again, this early establishment of memory pools was reversed by the addition of CpG. However, it is important to note that some level of inflammation is needed for T cell memory formation (Shaulov and Murali-Krishna, 2008). There is a substantial body of evidence that the inflammatory stimuli can affect the molecular signatures of the responding T cells. The expression of T-bet, as mentioned above, can clearly direct antigen-specific T cells into either a memory or an effector pathway (Joshi et al., 2007). Similarly, modulation of transcription factors such as Blimp-1 (Kallies et al., 2009; Rutishauser et al., 2009; Shin et al., 2009) and Eomes (Intlekofer et al., 2005) is necessary for the acquisition of effector versus memory lineage commitment. The main cytokines that affect the expression of theses transcription factors for responding T cells are IL-12, IL-2, IL-15, and IL-7, and the type-1 interferons. Dissecting the difference between the “good inflammation” necessary for the establishment of immunological memory versus the “bad inflammation” that leads to damaging immunopathology is imperative for future vaccine design.

## LONG-TERM PERSISTENCE OF T CELL MEMORY

### INSIGHTS GAINED FROM STUDIES IN AGED ANIMALS

Animal models have provided important insights into the persistence of T cell memory. In particular, experiments in aged animals allow a controlled analysis of the persistence of memory T cells over the lifetime that may follow an initial exposure to, and recovery from, a pathogenic infection. Such studies also allow us to study the recall of these memory cell populations after a subsequent pathogen challenge. Aged mice and non-human primates, similar to the elderly human population, are particularly susceptible to novel viral and bacterial infections, leading to an increase in the occurrence of severe disease. Thus, the impact of age on T cell responsiveness to infection or vaccination, together with the potential implications for both memory persistence into old age and the *de novo* generation of such memory in the elderly, are priority areas for analysis.

The reduced efficacy of T cell responses to infection and vaccination with age is thought to reflect a decline in both naïve T cell numbers and TCR diversity, together with the functional compromise of the “aged” naïve T cells. The thymic epithelium involutes with age, substantially reducing the generation and export of naïve T cells (Yunis et al., 1973; Simpson et al., 1975). Thus, maintenance of the naïve T cell pool becomes more dependent on homeostatic turnover, evidenced by clonal expansions in the naïve T cell pool (LeMaoult et al., 2000; Cicin-Sain et al., 2007; Ahmed et al., 2009) and the acquisition of a “memory-like” CD44^hi^ phenotype in older mice (Haluszczak et al., 2009; Rudd et al., 2011b; Decman et al., 2012). These time-associated alterations to the naïve T cell pool are accompanied by an age-related increase in the proportion of memory T cells due to pathogen encounter, which is consistent with reported expansions in the memory pool with immunological experience (Vezys et al., 2009). Moreover, large clonal expansions in the memory T cell repertoire associated with acute infection or primary vaccination (Cicin-Sain et al., 2007; Ely et al., 2007) and chronic infections are often observed (Ouyang et al., 2003; Ely et al., 2007; Ahmed et al., 2009; Kohlmeier et al., 2010). These factors collectively contribute to perturbations and a reduction in TCR diversity in the naïve T cell repertoire, which results in the preferential survival of high avidity TCR clonotypes (Cicin-Sain et al., 2007; Ahmed et al., 2009; Rudd et al., 2011b; Decman et al., 2012). As a consequence, aged individuals have impaired capacity to recruit an immune response a diverse array of TCR clonotypes when exposed to some novel infectious process (Yager et al., 2008; Rudd et al., 2011a; Bunztman et al., 2012; Valkenburg et al., 2012), but is instead dominated by clonal expansions or reduced diversity relative to younger counterparts.

Apart from a decline in the number and diversity of naïve T cells, the “aged” naïve T cells are thought to be functionally defective, thus compromising the generation and persistence of T cell memory in aged individuals. Recent reviews on age-related changes for CD4^+^ (Haynes and Swain, 2012) and CD8^+^ (Nikolich-Zugich et al., 2012) T cell responses summarize our understanding of such functional defects in antigen-specific T cell responses in the “elderly.” These phenotypically obvious defects include reductions in the response magnitude, the capacity to proliferate and differentiate following activation, narrowed T cell polyfunctionality, and the increase in expression in inhibitory receptors associated typically with functional exhaustion. We refer readers to these excellent reviews in order to focus the remainder of this discussion on our recent finding concerning the generation and persistence of influenza-specific CD8^+^ T cell memory in aged mice.

Previous mouse studies have established that aging is associated with diminished CD8^+^ T cell efficacy and delayed influenza virus clearance (Effros and Walford, 1983; Bender et al., 1991; Bender et al., 1995; Dong et al., 2000; Po et al., 2002). Recent evidence has further shown that the selective loss of primary, influenza-specific CD8^+^ T cell responsiveness in older mice is characterized by a narrowing in the spectrum of TCR usage that is seen predominantly for low frequency populations, with this effect being best characterized for the prominent D^b^NP_366_^+^ CD8^+^ T cell set (Yager et al., 2008; Toapanta and Ross, 2009; Valkenburg et al., 2012). Our work established that primary influenza virus infection of naïve 22-month (versus 12 week) old mice resulted in reduced numbers of immunodominant NP_366_^+^CD8^+^ and PA_224_^+^CD8^+^ T cells in the spleen compared with young adult mice (Valkenburg et al., 2012). In contrast, the difference in the magnitude for epitope-specific CD8^+^ T cell responses between young and old mice was not observed at the site of infection sampled by bronchoalveolar lavage (Valkenburg and Kedzierska, unpublished). This supports our previous findings that in the event of decreased T cell numbers, virus-specific CD8^+^ T cells traffic preferentially to the site of infection (Valkenburg et al., 2010). However, despite there being comparable CD8^+^ T cell counts at the site of infection for young and old, the “aged” “inflammatory” CD8^+^ T cells displayed impaired cytokine profiles (Valkenburg and Kedzierska, unpublished), similar to those found for the spleen (Valkenburg et al., 2010).

As a consequence, mice primed late (at 22 months) and then challenged even later in life (at 24 months) developed secondary CD8^+^ T cell responses that were diminished in quantity, quality, and TCR repertoire diversity (Valkenburg et al., 2012). This suggests that the memory CD8^+^ T cell pools established late in life are defective. Similarly, a recent study (Decman et al., 2010) suggested that infecting mice with LCMV or influenza at an extreme age (18–20 months) leads to defective CD8^+^ T cell memory and diminished recall responses following virus challenge. Thus, the age at initial priming is a critical determinant of CTL numbers, diversity, and function, with memory CD8^+^ T cell populations that are generated later in life being generally less efficacious (Eaton et al., 2008).

A key issue that then arises is whether we can recruit and maintain a pool of responsive T cells by priming those cells early in life, especially to diseases such as influenza, which are a particular threat to the elderly. We probed this question for influenza-specific CD8^+^ T cell memory. Our studies found that the ability to mount a CD8^+^ T cell response to influenza infection waned with age. However, early vaccination (at 6 weeks of age) prior to the attrition of low frequency anti-influenza CD8^+^ T cells was important to maintain the numbers, function, and a diverse array of TCRs of the memory pools for the life-time of an animal (Valkenburg et al., 2012). The TCR repertoire in extremely aged mice was “locked in” at an early age following vaccination, which proved advantageous for providing a high avidity and high magnitude response later in life. Consequently, memory T cells generated by influenza priming at a young age had a better protective capacity that was evidenced by accelerated viral clearance and reduced body weight loss in the aged animals as compared to the aged mice responding to primary infection (Valkenburg and Kedzierska, unpublished data). Hence, it is important to establish influenza-specific CD8^+^ T cell responses early in life to preserve optimal, T cell responsiveness and protect against the age-related attrition of naïve T cell precursors. These findings also suggest that designing influenza vaccines, which promote as-broad-as-possible spectrum of CD8^+^ T cell memory in adolescence could be beneficial, even if such benefit emerges long after the subject has first seen the protective immunogen.

### LONGEVITY OF HUMAN T CELL MEMORY

Novel insights into the longevity and function of human memory T cell responses have been provided by the elegant studies exploiting early live virus vaccination against yellow fever (YFV-17D) or smallpox (Dryvax; VV; reviewed in Ahmed and Akondy, 2011). Immunization with YFV-17 enabled analysis of YFV-specific T cell responses for individuals residing in countries where they would have had no exposure to wild-type infection. Using this approach, longitudinal analyses showed that a single dose of YFV-17D immunization elicits potent effector CD8^+^ and CD4^+^ T cell responses that can then be maintained as long-lasting memory populations (Co et al., 2002, 2009; Akondy et al., 2009; Wrammert et al., 2009). These memory sets had proportionally contracted from larger effector pools, co-incident to expression of CD127 (IL-7Rα; Akondy et al., 2009). Importantly, YFV-specific T cell pools could be detected decades after immunization (Ahmed and Akondy, 2011).

Similarly, exposure to vaccinia virus (VV), to protect against smallpox (or VV recombinants), has enabled the assessment of acute T cell responses and memory in the absence of secondary, “live virus” challenge (Walker and Slifka, 2010). In-depth analysis of memory T cell persistence suggests that the estimated half-life (t_1/2_) for CD8^+^ T cells is between 8 and 15 years, whereas t_1/2_ for the VV-specific CD4^+^ set is between 8 and 12 years (Hammarlund et al., 2003; Seder and Ahmed, 2003). At the peak of the acute VV-specific T cell responses, occurring within 2–3 weeks of human vaccination (Terajima et al., 2003; Kennedy et al., 2004; Miller et al., 2008), the numbers of VV-specific effector CD8^+^ T cells are higher than those of CD4^+^ T cells within the same individual (Amara et al., 2004; Miller et al., 2008). However, a preferential loss of VV-specific CD8^+^ T cell memory is experienced by ~50% of individuals within 20 years post-vaccination (Hammarlund et al., 2003). Interestingly, the t_1/2_ for T cells is much shorter than the t_1/2_ (92 years) for VV-specific antibody responses (Walker and Slifka, 2010). However, though the T cell t_1/2_ appears to be <16 years, VV-specific T cells can be detected in some individuals for up to 75 years by IFN-γ/TNF-α staining (Hammarlund et al., 2003). In comparison, measles virus (MV)-specific memory T cells induced after vaccination can be found for at least 34 years, with CD8^+^ T cell memory being more stable than CD4^+^ T cell memory (Naniche et al., 2004). Taken together, the above studies with YFV, VV and MV suggest a long-term persistence of human T cell memory that is in every sense comparable to the profiles analyzed experimentally in mice.

The persistence of human CD8^+^ T cell memory after influenza virus infection is also of a particular interest, given the commonplace recurrence and the observed susceptibility in elderly populations. In contrast to strain-specific antibodies, T cells elicit broader immunity to a spectrum of influenza strains (seasonal and pandemic) as they generally recognize more conserved, internal components of the virus. Both CD4^+^ and CD8^+^ T cell sets play an important role in influenza-specific T cell-mediated immunity (McMichael et al., 1983b; Wilkinson et al., 2012). The effector CD8^+^ T cells function to recognize and destroy influenza virus-infected cells. Pre-existing CD8^+^ T cell memory promotes more rapid recovery via the production of pro-inflammatory cytokines and direct killing of virus-producing cells (Doherty et al., 1996). Both experimental and epidemiological evidence indicates that memory CD8^+^ T cells primed by prior exposure to a seasonal influenza infection provide protection against subsequent challenge with novel, HA- and NA-distinct strains. Some virus replication still occurs, but the disease is less severe and the clinical outcome is improved. Evidence from animal models (Christensen et al., 2000) and humans (McMichael et al., 1983b; Epstein, 2006; Kreijtz et al., 2008; Lee et al., 2008) supports this protective role for CD8^+^ T-cells following heterologous priming and challenge between H1N1, H7N7, H3N2, and H5N1 influenza viruses. However, persistence of influenza-specific memory T cells in humans is understudied, although early study by McMichael et al. (1983a) used ^51^Cr-killing activity assay in PBMCs from individuals in 1977–1982 to infer contraction of T cell memory. It is, however, possible that a decrease in ^51^Cr lysis could be partially related to the loss of the killing capacity (Stambas et al., 2007) or cytolytic molecule expression (gzm A, gzm B) in the long-term memory CD8^+^ T cells (Jenkins et al., 2007) as shown previously by our studies in a mouse model of influenza infection. Similar observations have been made for human CD8^+^ T cells, with both granzyme A and granzyme B expression decreasing from 60% 1 month after infection to 33% within 1 year after infection (Rock et al., 2005). Further studies epitope-specific CD8^+^ T cells (across different HLAs) using the more recent tetramer technology is needed to further explore this issue of memory CD8^+^ T cell persistence, and acute T cell responses, after human influenza virus infection.

Our recent antigenic analysis of the 2009-H1N1 virus, showed that this newly emerged pandemic virus shared immunogenic peptides with the catastrophic 1918-H1N1 strain (Gras et al., 2010), and shared antigenic similarity with viruses present prior to 1945. This resulted in the detection of cross-reactive CD8^+^ T cell immunity between the H1N1-2009-infected donors and 1918 derived peptides for the NP_418_ epitope restricted by the large HLA-B7^+^ family. Cross-reactive immunity between the pandemic 2009-H1N1 strain and the 1918-H1N1 strain (as well as other H1N1 viruses from the beginning of the century) at both T cell and antibody levels may have resulted in lower susceptibility to the H1N1-2009 in individuals >65 years of age, with the majority of severe cases occurring in young adults (the mean patient age was 24 years; Cao et al., 2009; Agrati et al., 2010). This was in contrast to the usual scenario of the elderly population being more susceptible to annual, seasonal epidemics caused by influenza A viruses (Couch et al., 1986; Webster, 2000). Therefore, the lack of T cell immunity in younger adults and children, and the persistence of cross-reactive memory T cells in older individuals may partially account for the demographics of infection. The question thus still remains whether memory CD8^+^ T cell populations to influenza strains encountered early in life persist for a life-time.

A previous report on CD8^+^ T cell responses in elderly individuals showed no difference in the frequency of influenza-specific CTLs between the 18 and 70 years-old cohort, however there was lower lytic capacity in the 68–70 years versus the 18–20-year-old donors (Boon et al., 2002). Then, while there were no significant differences in IFN-γ^+^ CD3^+^ CD8^+^ or CD4^+^ T cell numbers with age, the proportion of IL-4^+^ CD3^+^ CD8^+^ and IFN-γ^+^ CD3^+^ CD8^+^ (detected following PBMC stimulation with PMA/inomycin) increased in the elderly. Furthermore, the T cell proliferative response was significantly higher in the 18–20 years-old cohort, and was not antigen-dose dependent. That is, increasing antigen dose did not compensate for the reduced PBMC proliferation in 68–70 years-old individuals.

Interestingly, CD45RO (memory) and CD45RA (naïve) expression varied between different age groups. In the 18- to 20-year-old cohort, the prevalence of CD45RO versus CD45RA expression was similar for CD3^+^CD4^+^ T cells, while a CD45RA > CD45RO distribution was found for the CD3^+^CD8^+^ set. By contrast, the elderly (Ouyang et al., 2003; Yager et al., 2008; Kohlmeier et al., 2010) group showed a CD45RO > CD45RA profile for both CD3^+^CD4^+^ and CD3^+^CD8^+^ T cells, together with reduced CD28 expression (for both CD4^+^ and CD8^+^ T cells) when compared with the youngsters. Furthermore, there was a trend for lower lytic capacity in the age 68–70 set following *in vitro* PBMC stimulation (Boon et al., 2002). This could be related to lower levels of perforin/granzyme expression. Overall, the analysis to date suggests that influenza-specific CD8^+^ T cells persist, though their cytolytic- and cytokine-producing potential may decline with increasing years. But the findings so far are limited in both scope and sophistication. There is clearly a need for much more detailed analysis of aging, influenza-specific human T cells, from the aspects of numbers, function, repertoire diversity, and capacity for effective recall.

## SUMMARY

The future design of T cell-based vaccination strategies that can provide effective and optimal protection across the lifespan of an individual crucially depends upon an in-depth understanding of the development and maintenance of T cell memory and the factors that impact the protective capacity of memory T cell populations. Studies in recent years have made substantial progress in dissecting the complexities of T cell memory populations, resulting in the identification of major factors that influence the composition and stability of these T cell memory populations. While these advances have moved us closer to elucidating the mechanisms contributing to optimal T cell memory generation, there remain many aspects of T cell memory to be investigated. Studies continue to reveal increased phenotypic, functional, and anatomical heterogeneity of T cell memory populations. The temporal changes to the composition and protective abilities of these T cell memory subsets require additional study in order to determine what constitutes optimal T cell memory. An important consideration in future investigations of T cell memory is the increasing evidence that the protective abilities of memory T cells are dependent on the pathogen and the nature of the infection. One potential complicating factor in the maintenance of life-long immunity is the changes at the cellular, environmental, and population level of T cells that occur in later life that have been associated with impaired immune responsiveness in the elderly. However, studies to date indicate that these age-related defects in T cell responses primarily affect the generation of T cell memory in old age. This suggests that to address the increased immune susceptibility in the elderly will require either the development of vaccines to be administered earlier in life, to generate T cell memory that will provide protection the against those pathogens likely to be encountered in later life, or the development of strategies to prevent or treat age-related defects in T cell immunity. Either approach provides substantial challenges for immunological research in terms of improving our understanding of temporal changes to T cell immunity.

## Conflict of Interest Statement

The authors declare that the research was conducted in the absence of any commercial or financial relationships that could be construed as a potential conflict of interest.

## References

[B1] Agrati C. Gioia C. Lalle E. Cimini E. Castilletti C. Armignacco O. (2010). Association of profoundly impaired immune competence in H1N1v-infected patients with a severe or fatal clinical course. *J. Infect. Dis.* 202 681–689 20670171 10.1086/655469

[B2] Ahmed M. Lanzer K. G. Yager E. J. Adams P. S. Johnson L. L. Blackman M. A. (2009). Clonal expansions and loss of receptor diversity in the naive CD8 T cell repertoire of aged mice. *J. Immunol.* 182 784–792 19124721 10.4049/jimmunol.182.2.784 PMC2724652

[B3] Ahmed R. Akondy R. S. (2011). Insights into human CD8(+) T-cell memory using the yellow fever and smallpox vaccines. *Immunol. Cell Biol.* 89 340–345 21301482 10.1038/icb.2010.155

[B4] Akondy R. S. Monson N. D. Miller J. D. Edupuganti S. Teuwen D. Wu H. (2009). The yellow fever virus vaccine induces a broad and polyfunctional human memory CD8^+^ T cell response. *J. Immunol.* 183 7919–7930 19933869 10.4049/jimmunol.0803903 PMC3374958

[B5] Amara R. R. Nigam P. Sharma S. Liu J. Bostik V. (2004). Long-lived poxvirus immunity, robust CD4 help, and better persistence of CD4 than CD8 T cells. *J. Virol.* 78 3811–3816 15047796 10.1128/JVI.78.8.3811-3816.2004 PMC374286

[B6] Badovinac V. P. Messingham K. A. Griffith T. S. Harty J. T. (2006). TRAIL deficiency delays, but does not prevent, erosion in the quality of “helpless” memory CD8 T cells. *J. Immunol.* 177 999–1006 16818756 10.4049/jimmunol.177.2.999

[B7] Badovinac V. P. Messingham K. A. Hamilton S. E. Harty J. T. (2003). Regulation of CD8^+^ T cells undergoing primary and secondary responses to infection in the same host. *J. Immunol.* 170 4933–4942 12734336 10.4049/jimmunol.170.10.4933

[B8] Badovinac V. P. Messingham K. A. Jabbari A. Haring J. S. Harty J. T. (2005). Accelerated CD8^+^ T-cell memory and prime-boost response after dendritic-cell vaccination. *Nat. Med.* 11 748–756 15951824 10.1038/nm1257

[B9] Badovinac V. P. Porter B. B. Harty J. T. (2004). CD8^+^ T cell contraction is controlled by early inflammation. *Nat. Immunol.* 5 809–817 15247915 10.1038/ni1098

[B10] Baron V. Bouneaud C. Cumano A. Lim A. Arstila T. P. Kourilsky P. (2003). The repertoires of circulating human CD8(+) central and effector memory T cell subsets are largely distinct. *Immunity* 18 193–204 12594947 10.1016/s1074-7613(03)00020-7

[B11] Belz G. T. Masson F. (2010). Interleukin-2 tickles T cell memory. *Immunity* 32 7–9 20152165 10.1016/j.immuni.2010.01.009

[B12] Belz G. T. Wodarz D. Diaz G. Nowak M. A. Doherty P. C. (2002). Compromised influenza virus-specific CD8(+)-T-cell memory in CD4(+)-T-cell-deficient mice. *J. Virol.* 76 12388–12393 12414983 10.1128/JVI.76.23.12388-12393.2002 PMC136883

[B13] Belz G. T. Xie W. Altman J. D. Doherty P. C. (2000). A previously unrecognized H-2D(b)-restricted peptide prominent in the primary influenza A virus-specific CD8(+) T-cell response is much less apparent following secondary challenge. *J. Virol.* 74 3486–3493 10729122 10.1128/jvi.74.8.3486-3493.2000 PMC111856

[B14] Bender B. S. Johnson M. P. Small P. A. (1991). Influenza in senescent mice: impaired cytotoxic T-lymphocyte activity is correlated with prolonged infection. *Immunology* 72 514–519 2037313 PMC1384370

[B15] Bender B. S. Taylor S. F. Zander D. S. Cottey R. (1995). Pulmonary immune response of young and aged mice after influenza challenge. *J. Lab. Clin. Med.* 126 169–177 7636390

[B16] Boon A. C. Fringuelli E. Graus Y. M. Fouchier R. A. Sintnicolaas K. Iorio A. M. (2002). Influenza A virus specific T cell immunity in humans during aging. *Virology* 299 100–108 12167345 10.1006/viro.2002.1491

[B17] Bouneaud C. Garcia Z. Kourilsky P. Pannetier C. (2005). Lineage relationships, homeostasis, and recall capacities of central- and effector-memory CD8 T cells in vivo. *J. Exp. Med.* 201 579–590 15710650 10.1084/jem.20040876 PMC2213051

[B18] Bunztman A. Vincent B. G. Krovi H. Steele S. Frelinger J. A. (2012). The LCMV gp33-specific memory T cell repertoire narrows with age. *Immun. Ageing* 9 17 10.1186/1742-4933-9-17 PMC3472190 22894656

[B19] Cao B. Li X. W. Mao Y. Wang J. Lu H. Z. Chen Y. S. (2009). Clinical features of the initial cases of 2009 pandemic influenza A (H1N1) virus infection in China. *N. Engl. J. Med.* 361 2507–2517 20007555 10.1056/NEJMoa0906612

[B20] Christensen J. P. Doherty P. C. Branum K. C. Riberdy J. M. (2000). Profound protection against respiratory challenge with a lethal H7N7 influenza A virus by increasing the magnitude of CD8(+) T-cell memory. *J. Virol.* 74 11690–11696 11090168 10.1128/jvi.74.24.11690-11696.2000 PMC112451

[B21] Cicin-Sain L. Messaoudi I. Park B. Currier N. Planer S. Fischer M. (2007). Dramatic increase in naive T cell turnover is linked to loss of naive T cells from old primates. *Proc. Natl. Acad. Sci. U.S.A.* 104 19960–19965 18056811 10.1073/pnas.0705905104 PMC2148405

[B22] Co M. D. Kilpatrick E. D. Rothman A. L. (2009). Dynamics of the CD8 T-cell response following yellow fever virus 17D immunization. *Immunology* 128 e718–e727 19740333 10.1111/j.1365-2567.2009.03070.x PMC2753898

[B23] Co M. D. Terajima M. Cruz J. Ennis F. A. Rothman A. L. (2002). Human cytotoxic T lymphocyte responses to live attenuated 17D yellow fever vaccine: identification of HLA-B35-restricted CTL epitopes on nonstructural proteins NS1, NS2b, NS3, and the structural protein E. *Virology* 293 151–163 11853408 10.1006/viro.2001.1255

[B24] Couch R. B. Kasel J. A. Glezen W. P. Cate T. R. Six H. R. Taber L. H. (1986). Influenza: its control in persons and populations. *J. Infect. Dis.* 153 431–440 3950437 10.1093/infdis/153.3.431

[B25] Croom H. A. Denton A. E. Valkenburg S. A. Swan N. G. Olson M. R. Turner S. J. (2011). Memory precursor phenotype of CD8^+^ T cells reflects early antigenic experience rather than memory numbers in a model of localized acute influenza infection. *Eur. J. Immunol.* 41 682–693 21264852 10.1002/eji.201040625

[B26] Decman V. Laidlaw B. J. Dimenna L. J. Abdulla S. Mozdzanowska K. Erikson J. (2010). Cell-intrinsic defects in the proliferative response of antiviral memory CD8 T cells in aged mice upon secondary infection. *J. Immunol.* 184 5151–5159 20368274 10.4049/jimmunol.0902063

[B27] Decman V. Laidlaw B. J. Doering T. A. Leng J. Ertl H. C. Goldstein D. R. (2012). Defective CD8 T cell responses in aged mice are due to quantitative and qualitative changes in virus-specific precursors. *J. Immunol.* 188 1933–1941 22246631 10.4049/jimmunol.1101098 PMC3320034

[B28] Doherty P. C. Christensen J. P. (2000). Accessing complexity: the dynamics of virus-specific T cell responses. *Annu. Rev. Immunol.* 18 561–592 10837069 10.1146/annurev.immunol.18.1.561

[B29] Doherty P. C. Topham D. J. Tripp R. A. (1996). Establishment and persistence of virus-specific CD4^+^ and CD8^+^ T cell memory. *Immunol. Rev.* 150 23–44 8782700 10.1111/j.1600-065x.1996.tb00694.x

[B30] Dong L. Mori I. Hossain M. J. Kimura Y. (2000). The senescence-accelerated mouse shows aging-related defects in cellular but not humoral immunity against influenza virus infection. *J. Infect. Dis.* 182 391–396 10915067 10.1086/315727

[B31] Eaton S. M. Maue A. C. Swain S. L. Haynes L. (2008). Bone marrow precursor cells from aged mice generate CD4 T cells that function well in primary and memory responses. *J. Immunol.* 181 4825–4831 18802086 10.4049/jimmunol.181.7.4825 PMC2582190

[B32] Effros R. B. Walford R. L. (1983). Diminished T-cell response to influenza virus in aged mice. *Immunology* 49 387–392 6602091 PMC1454174

[B33] Ely K. H. Ahmed M. Kohlmeier J. E. Roberts A. D. Wittmer S. T. Blackman M. A. (2007). Antigen-specific CD8^+^ T cell clonal expansions develop from memory T cell pools established by acute respiratory virus infections. *J. Immunol.* 179 3535–3542 17785787 10.4049/jimmunol.179.6.3535

[B34] Epstein S. L. (2006). Prior H1N1 influenza infection and susceptibility of Cleveland Family Study participants during the H2N2 pandemic of 1957: an experiment of nature. *J. Infect. Dis.* 193 49–53 16323131 10.1086/498980

[B35] Gebhardt T. Wakim L. M. Eidsmo L. Reading P. C. Heath W. R. Carbone F. R. (2009). Memory T cells in nonlymphoid tissue that provide enhanced local immunity during infection with herpes simplex virus. *Nat. immunol.* 10 524–530 19305395 10.1038/ni.1718

[B36] Gras S. Kedzierski L. Valkenburg S. A. Laurie K. Liu Y. C. Denholm J. T. (2010). Cross-reactive CD8^+^ T-cell immunity between the pandemic H1N1-2009 and H1N1-1918 influenza A viruses. *Proc. Natl. Acad. Sci. U.S.A.* 107 12599–12604 20616031 10.1073/pnas.1007270107 PMC2906563

[B37] Haluszczak C. Akue A. D. Hamilton S. E. Johnson L. D. Pujanauski L. Teodorovic L. (2009). The antigen-specific CD8^+^ T cell repertoire in unimmunized mice includes memory phenotype cells bearing markers of homeostatic expansion. *J. Exp. Med.* 206 435–448 19188498 10.1084/jem.20081829 PMC2646575

[B38] Hammarlund E. Lewis M. W. Hansen S. G. Strelow L. I. Nelson J. A. Sexton G. J. (2003). Duration of antiviral immunity after smallpox vaccination. *Nat. Med.* 9 1131–1137 12925846 10.1038/nm917

[B39] Hand T. W. Morre M. Kaech S. M. (2007). Expression of IL-7 receptor alpha is necessary but not sufficient for the formation of memory CD8 T cells during viral infection. *Proc. Natl. Acad. Sci. U.S.A.* 104 11730–11735 17609371 10.1073/pnas.0705007104 PMC1913873

[B40] Haynes L. Swain S. L. (2012). Aged-related shifts in T cell homeostasis lead to intrinsic T cell defects. *Semin. Immunol.* 24 350–355 22564707 10.1016/j.smim.2012.04.001 PMC3415577

[B41] Hikono H. Kohlmeier J. E. Takamura S. Wittmer S. T. Roberts A. D. Woodland D. L. (2007). Activation phenotype, rather than central- or effector-memory phenotype, predicts the recall efficacy of memory CD8^+^ T cells. *J. Exp. Med.* 204 1625–1636 17606632 10.1084/jem.20070322 PMC2118640

[B42] Hogan R. J. Usherwood E. J. Zhong W. Roberts A. A. Dutton R. W. Harmsen A. G. (2001). Activated antigen-specific CD8^+^ T cells persist in the lungs following recovery from respiratory virus infections. *J. Immunol.* 166 1813–1822 11160228 10.4049/jimmunol.166.3.1813

[B43] Huster K. M. Busch V. Schiemann M. Linkemann K. Kerksiek K. M. Wagner H. (2004). Selective expression of IL-7 receptor on memory T cells identifies early CD40L-dependent generation of distinct CD8^+^ memory T cell subsets. *Proc. Natl. Acad. Sci. U.S.A.* 101 5610–5615 15044705 10.1073/pnas.0308054101 PMC397444

[B44] Intlekofer A. M. Takemoto N. Wherry E. J. Longworth S. A. Northrup J. T. Palanivel V. R. (2005). Effector and memory CD8^+^ T cell fate coupled by T-bet and eomesodermin. *Nat. Immunol.* 6 1236–1244 16273099 10.1038/ni1268

[B45] Janssen E. M. Droin N. M. Lemmens E. E. Pinkoski M. J. Bensinger S. J. Ehst B. D. (2005). CD4^+^ T-cell help controls CD8^+^ T-cell memory via TRAIL-mediated activation-induced cell death. *Nature* 434 88–93 15744305 10.1038/nature03337

[B46] Jenkins M. R. Kedzierska K. Doherty P. C. Turner S. J. (2007). Heterogeneity of effector phenotype for acute phase and memory influenza A virus-specific CTL. *J. Immunol.* 179 64–70 17579022 10.4049/jimmunol.179.1.64

[B47] Joshi N. S. Cui W. Chandele A. Lee H. K. Urso D. R. Hagman J. (2007). Inflammation directs memory precursor and short-lived effector CD8(+) T cell fates via the graded expression of T-bet transcription factor. *Immunity* 27 281–295 17723218 10.1016/j.immuni.2007.07.010 PMC2034442

[B48] Kaech S. M. Ahmed R. (2001). Memory CD8^+^ T cell differentiation: initial antigen encounter triggers a developmental program in naive cells. *Nat. Immunol.* 2 415–422 11323695 10.1038/87720 PMC3760150

[B49] Kaech S. M. Tan J. T. Wherry E. J. Konieczny B. T. Surh C. D. Ahmed R. (2003). Selective expression of the interleukin 7 receptor identifies effector CD8 T cells that give rise to long-lived memory cells. *Nat. Immunol.* 4 1191–1198 14625547 10.1038/ni1009

[B50] Kalia V. Sarkar S. Subramaniam S. Haining W. N. Smith K. A. Ahmed R. (2010). Prolonged interleukin-2Ralpha expression on virus-specific CD8^+^ T cells favors terminal-effector differentiation *in vivo*. *Immunity* 32 91–103 20096608 10.1016/j.immuni.2009.11.010

[B51] Kallies A. Xin A. Belz G. T. Nutt S. L. (2009). Blimp-1 transcription factor is required for the differentiation of effector CD8(+) T cells and memory responses. *Immunity* 31 283–295 19664942 10.1016/j.immuni.2009.06.021

[B52] Kedzierska K. Stambas J. Jenkins M. R. Keating R. Turner S. J. Doherty P. C. (2007). Location rather than CD62L phenotype is critical in the early establishment of influenza-specific CD8+ T cell memory. *Proc. Natl. Acad. Sci. U.S.A.* 104 9782–9787 17522251 10.1073/pnas.0703699104 PMC1887603

[B53] Kedzierska K. Venturi V. Field K. Davenport M. P. Turner S. J. Doherty P. C. (2006). Early establishment of diverse TCR profiles for influenza-specific CD62L^hi^ CD8^+^ memory T cells. *Proc. Natl. Acad. Sci. U.S.A.* 103 9184–9189 16754852 10.1073/pnas.0603289103 PMC1482587

[B54] Kedzierska K. Venturi V. Valkenburg S. A. Davenport M. P. Turner S. J. Doherty P. C. (2008). Homogenization of TCR repertoires within secondary CD62Lhigh and CD62Llow virus-specific CD8+ T cell populations. *J. Immunol.* 180 7938–7947 18523257 10.4049/jimmunol.180.12.7938

[B55] Kennedy J. S. Frey S. E. Yan L. Rothman A. L. Cruz J. Newman F. K. (2004). Induction of human T cell-mediated immune responses after primary and secondary smallpox vaccination. *J. Infect. Dis.* 190 1286–1294 15346340 10.1086/423848

[B56] Kohlmeier J. E. Connor L. M. Roberts A. D. Cookenham T. Martin K. Woodland D. L. (2010). Nonmalignant clonal expansions of memory CD8^+^ T cells that arise with age vary in their capacity to mount recall responses to infection. *J. Immunol.* 185 3456–3462 20720204 10.4049/jimmunol.1001745 PMC2933333

[B57] Kreijtz J. H. de Mutsert G. van Baalen C. A. Fouchier R. A. Osterhaus A. D. Rimmelzwaan G. F. (2008). Cross-recognition of avian H5N1 influenza virus by human cytotoxic T lymphocyte populations directed to human influenza A virus. *J. Virol.* 82 5161–5166 18353950 10.1128/JVI.02694-07 PMC2395172

[B58] La Gruta N. L. Rothwell W. T. Cukalac T. Swan N. G. Valkenburg S. A. Kedzierska K. (2010). Primary CTL response magnitude in mice is determined by the extent of naive T cell recruitment and subsequent clonal expansion. *J. Clin. Invest.* 120 1885–1894 20440073 10.1172/JCI41538 PMC2877949

[B59] Lee L. Y. Ha do L. A. Simmons C. de Jong M. D. Chau N. V. Schumacher R. (2008). Memory T cells established by seasonal human influenza A infection cross-react with avian influenza A (H5N1) in healthy individuals. *J. Clin. Invest.* 118 3478–3490 18802496 10.1172/JCI32460 PMC2542885

[B60] LeMaoult J. Messaoudi I. Manavalan J. S. Potvin H. Nikolich-Zugich D. Dyall R. (2000). Age-related dysregulation in CD8 T cell homeostasis: kinetics of a diversity loss. *J. Immunol.* 165 2367–2373 10946259 10.4049/jimmunol.165.5.2367

[B61] Mackay L. K. Stock A. T. Ma J. Z. Jones C. M. Kent S. J. Mueller S. N. (2012). Long-lived epithelial immunity by tissue-resident memory T (TRM) cells in the absence of persisting local antigen presentation. *Proc. Natl. Acad. Sci. U.S.A.* 109 7037–7042 22509047 10.1073/pnas.1202288109 PMC3344960

[B62] Marshall D. R. Turner S. J. Belz G. T. Wingo S. Andreansky S. Sangster M. Y. (2001). Measuring the diaspora for virus-specific CD8^+^ T cells. *Proc. Natl. Acad. Sci. U.S.A.* 98 6313–6318 11344265 10.1073/pnas.101132698 PMC33465

[B63] Masopust D. Vezys V. Marzo A. L. Lefrancois L. (2001). Preferential localization of effector memory cells in nonlymphoid tissue. *Science* 291 2413–2417 11264538 10.1126/science.1058867

[B64] McKinstry K. K. Strutt T. M. Kuang Y. Brown D. M. Sell S. Dutton, R. W., et al. (2012). Memory CD4^+^ T cells protect against influenza through multiple synergizing mechanisms. *J. Clin. Invest.* 122 2847–2856 22820287 10.1172/JCI63689 PMC3408751

[B65] McMichael A. J. Gotch F. M. Dongworth D. W. Clark A. Potter C. W. (1983a). Declining T-cell immunity to influenza, 1977-82. *Lancet* 2 762–764 6137602 10.1016/s0140-6736(83)92297-3

[B66] McMichael A. J. Gotch F. M. Noble G. R. Beare P. A. (1983b). Cytotoxic T-cell immunity to influenza. *N. Engl. J. Med.* 309 13–17 6602294 10.1056/NEJM198307073090103

[B67] Miller J. D. van der Most R. G. Akondy R. S. Glidewell J. T. Albott S. Masopust D. (2008). Human effector and memory CD8^+^ T cell responses to smallpox and yellow fever vaccines. *Immunity* 28 710–722 18468462 10.1016/j.immuni.2008.02.020

[B68] Moon J. J. Chu H. H. Pepper M. McSorley S. J. Jameson S. C. Kedl R. M. (2007). Naive CD4(+) T cell frequency varies for different epitopes and predicts repertoire diversity and response magnitude. *Immunity* 27 203–213 17707129 10.1016/j.immuni.2007.07.007 PMC2200089

[B69] Naniche D. Garenne M. Rae C. Manchester M. Buchta R. Brodine S. K. (2004). Decrease in measles virus-specific CD4 T cell memory in vaccinated subjects. *J. Infect. Dis.* 190 1387–1395 15378430 10.1086/424571

[B70] Nikolich-Zugich J. Li G. Uhrlaub J. L. Renkema K. R. Smithey M. J. (2012). Age-related changes in CD8 T cell homeostasis and immunity to infection. *Semin. Immunol.* 24 356–364 22554418 10.1016/j.smim.2012.04.009 PMC3480557

[B71] Nolz J. C. Harty J. T. (2011). Protective capacity of memory CD8^+^ T cells is dictated by antigen exposure history and nature of the infection. *Immunity* 34 781–793 21549619 10.1016/j.immuni.2011.03.020 PMC3103642

[B72] Obar J. J. Lefrancois L. (2010). Early signals during CD8(+) T cell priming regulate the generation of central memory cells. *J. Immunol.* 185 263–272 20519649 10.4049/jimmunol.1000492 PMC2997352

[B73] Ouyang Q. Wagner W. M. Walter S. Muller C. A. Wikby A. Aubert G. (2003). An age-related increase in the number of CD8^+^ T cells carrying receptors for an immunodominant Epstein–Barr virus (EBV) epitope is counteracted by a decreased frequency of their antigen-specific responsiveness. *Mech. Ageing Dev.* 124 477–485 12714256 10.1016/s0047-6374(03)00026-5

[B74] Pham N. L. Badovinac V. P. Harty J. T. (2009). A default pathway of memory CD8 T cell differentiation after dendritic cell immunization is deflected by encounter with inflammatory cytokines during antigen-driven proliferation. *J. Immunol.* 183 2337–2348 19635915 10.4049/jimmunol.0901203 PMC2786780

[B75] Pipkin M. E. Sacks J. A. Cruz-Guilloty F. Lichtenheld M. G. Bevan M. J. Rao A. (2010). Interleukin-2 and inflammation induce distinct transcriptional programs that promote the differentiation of effector cytolytic T cells. *Immunity* 32 79–90 20096607 10.1016/j.immuni.2009.11.012 PMC2906224

[B76] Po J. L. Gardner E. M. Anaraki F. Katsikis P. D. Murasko D. M. (2002). Age-associated decrease in virus-specific CD8^+^ T lymphocytes during primary influenza infection. *Mech. Ageing Dev.* 123 1167–1181 12044966 10.1016/s0047-6374(02)00010-6

[B77] Powell T. J. Strutt T. Reome J. Hollenbaugh J. A. Roberts A. D. Woodland D. L. (2007). Priming with cold-adapted influenza A does not prevent infection but elicits long-lived protection against supralethal challenge with heterosubtypic virus. *J. Immunol.* 178 1030–1038 17202366 10.4049/jimmunol.178.2.1030

[B78] Reinhardt R. L. Khoruts A. Merica R. Zell T. Jenkins M. K. (2001). Visualizing the generation of memory CD4 T cells in the whole body. *Nature* 410 101–105 11242050 10.1038/35065111

[B79] Riberdy J. M. Christensen J. P. Branum K. Doherty P. C. (2000). Diminished primary and secondary influenza virus-specific CD8(+) T-cell responses in CD4-depleted Ig(-/-) mice. *J. Virol.* 74 9762–9765 11000251 10.1128/jvi.74.20.9762-9765.2000 PMC112411

[B80] Roberts A. D. Woodland D. L. (2004). Cutting edge: effector memory CD8^+^ T cells play a prominent role in recall responses to secondary viral infection in the lung. *J. Immunol.* 172 6533–6537 15153466 10.4049/jimmunol.172.11.6533

[B81] Roberts A. D. Ely K. H. Woodland D. L. (2005). Differential contributions of central and effector memory T cells to recall responses. *J. Exp. Med.* 202 123–133 15983064 10.1084/jem.20050137 PMC2212898

[B82] Rock M. T. Yoder S. M. Wright P. F. Talbot T. R. Edwards K. M. Crowe J. E. Jr (2005). Differential regulation of granzyme and perforin in effector and memory T cells following smallpox immunization. *J. Immunol.* 174 3757–3764 15749916 10.4049/jimmunol.174.6.3757

[B83] Rudd B. D. Venturi V. Davenport M. P. Nikolich-Zugich J. (2011a). Evolution of the antigen-specific CD8^+^ TCR repertoire across the life span: evidence for clonal homogenization of the old TCR repertoire. *J. Immunol.* 186 2056–2064 21248263 10.4049/jimmunol.1003013 PMC4119821

[B84] Rudd B. D. Venturi V. Li G. Samadder P. Ertelt J. M. Way S. S. (2011b). Nonrandom attrition of the naive CD8^+^ T-cell pool with aging governed by T-cell receptor:pMHC interactions. *Proc. Natl. Acad. Sci. U.S.A.* 108 13694–13699 21813761 10.1073/pnas.1107594108 PMC3158207

[B85] Rutishauser R. L. Martins G. A. Kalachikov S. Chandele A. Parish I. A. Meffre E. (2009). Transcriptional repressor Blimp-1 promotes CD8(+) T cell terminal differentiation and represses the acquisition of central memory T cell properties. *Immunity* 31 296–308 19664941 10.1016/j.immuni.2009.05.014 PMC2783637

[B86] Sallusto F. Lenig D. Forster R. Lipp M. Lanzavecchia A. (1999). Two subsets of memory T lymphocytes with distinct homing potentials and effector functions. *Nature* 401 708–712 10537110 10.1038/44385

[B87] Schlub T. E. Badovinac V. P. Sabel J. T. Harty J. T. Davenport M. P. (2010). Predicting CD62L expression during the CD8^+^ T-cell response *in vivo*. *Immunol. Cell Biol.* 88 157–164 19859082 10.1038/icb.2009.80 PMC2824781

[B88] Schmidt N. W. Podyminogin R. L. Butler N. S. Badovinac V. P. Tucker B. J. Bahjat K. S. (2008). Memory CD8 T cell responses exceeding a large but definable threshold provide long-term immunity to malaria. *Proc. Natl. Acad. Sci. U.S.A.* 105 14017–14022 18780790 10.1073/pnas.0805452105 PMC2544571

[B89] Seder R. A. Ahmed R. (2003). Similarities and differences in CD4^+^ and CD8^+^ effector and memory T cell generation. *Nat. Immunol.* 4 835–842 12942084 10.1038/ni969

[B90] Shaulov A. Murali-Krishna K. (2008). CD8 T cell expansion and memory differentiation are facilitated by simultaneous and sustained exposure to antigenic and inflammatory milieu. *J. Immunol.* 180 1131–1138 18178853 10.4049/jimmunol.180.2.1131

[B91] Shin H. Blackburn S. D. Intlekofer A. M. Kao C. Angelosanto J. M. Reiner S. L. (2009). A role for the transcriptional repressor Blimp-1 in CD8(+) T cell exhaustion during chronic viral infection. *Immunity* 31 309–320 19664943 10.1016/j.immuni.2009.06.019 PMC2747257

[B92] Simpson J. G. Gray E. S. Beck J. S. (1975). Age involution in the normal human adult thymus. *Clin. Exp. Immunol.* 19 261–265 1212800 PMC1538100

[B93] Stambas J. Doherty P. C. Turner S. J. (2007). An *in vivo* cytotoxicity threshold for influenza a virus-specific effector and memory CD8^+^ T cells. *J. Immunol.* 178 1285–1292 17237374 10.4049/jimmunol.178.3.1285

[B94] Swain S. L. McKinstry K. K. Strutt T. M. (2012). Expanding roles for CD4(+) T cells in immunity to viruses. *Nat. Rev. Immunol.* 12 136–148 22266691 10.1038/nri3152 PMC3764486

[B95] Terajima M. Cruz J. Raines G. Kilpatrick E. D. Kennedy J. S. Rothman A. L. (2003). Quantitation of CD8^+^ T cell responses to newly identified HLA-A*0201-restricted T cell epitopes conserved among vaccinia and variola (smallpox) viruses. *J. Exp. Med.* 197 927–932 12668642 10.1084/jem.20022222 PMC2193889

[B96] Toapanta F. R. Ross T. M. (2009). Impaired immune responses in the lungs of aged mice following influenza infection. *Respir. Res.* 10 112 10.1186/1465-9921-10-112 PMC2785782 19922665

[B97] Topham D. J. Tripp R. A. Sarawar S. R. Sangster M. Y. Doherty P. C. (1996). Immune CD4^+^ T cells promote the clearance of influenza virus from major histocompatibility complex class II-/- respiratory epithelium. *J. Virol.* 70 1288–1291 8551597 10.1128/jvi.70.2.1288-1291.1996 PMC189945

[B98] Tripp R. A. Hou S. Doherty P. C. (1995). Temporal loss of the activated L-selectin-low phenotype for virus-specific CD8^+^ memory T cells. *J. Immunol.* 154 5870–5875 7538535

[B99] Valkenburg S. A. Gras S. Guillonneau C. La Gruta N. L. Thomas P. G. Purcell A. W. (2010). Protective efficacy of cross-reactive CD8^+^ T cells recognising mutant viral epitopes depends on peptide-MHC-I structural interactions and T cell activation threshold. *PLoS Pathog.* 6 e1001039 10.1371/journal.ppat.1001039 PMC2920842 20711359

[B100] Valkenburg S. A. Venturi V. Dang T. H. Bird N. L. Doherty P. C. Turner S. J. (2012). Early priming minimizes the age-related immune compromise of CD8 T cell diversity and function. *PLoS Pathog.* 8 e1002544 10.1371/journal.ppat.1002544 PMC3285595 22383879

[B101] Vezys V. Yates A. Casey K. A. Lanier G. Ahmed R. Antia R. (2009). Memory CD8 T-cell compartment grows in size with immunological experience. *Nature* 457 196–199 19005468 10.1038/nature07486

[B102] Wakim L. M. Woodward-Davis A. Liu R. Hu Y. Villadangos J. Smyth G. (2012). The molecular signature of tissue resident memory CD8 T cells isolated from the brain. *J. Immunol.* 189 3462–3471 22922816 10.4049/jimmunol.1201305 PMC3884813

[B103] Walker J. M. Slifka M. K. (2010). Longevity of T-cell memory following acute viral infection. *Adv. Exp. Med. Biol.* 684 96–107 20795543 10.1007/978-1-4419-6451-9_8

[B104] Webster R. G. (2000). Immunity to influenza in the elderly. *Vaccine* 18 1686–1689 10689149 10.1016/s0264-410x(99)00507-1

[B105] Wherry E. J. Becker T. C. Boone D. Kaja M. K. Ma A. Ahmed R. (2002). Homeostatic proliferation but not the generation of virus specific memory CD8 T cells is impaired in the absence of IL-15 or IL-15Ralpha. *Adv. Exp. Med. Biol.* 512 165–175 12405201 10.1007/978-1-4615-0757-4_22

[B106] Wherry E. J. Teichgraber V. Becker T. C. Masopust D. Kaech S. M. Antia R. (2003). Lineage relationship and protective immunity of memory CD8 T cell subsets. *Nat. Immunol.* 4 225–234 12563257 10.1038/ni889

[B107] Wilkinson T. M. Li C. K. Chui C. S. Huang A. K. Perkins M. Liebner J. C. (2012). Preexisting influenza-specific CD4^+^ T cells correlate with disease protection against influenza challenge in humans. *Nat. Med.* 18 274–280 22286307 10.1038/nm.2612

[B108] Wrammert J. Miller J. Akondy R. Ahmed R. (2009). Human immune memory to yellow fever and smallpox vaccination. *J. Clin. Immunol.* 29 151–157 19052852 10.1007/s10875-008-9267-3

[B109] Yager E. J. Ahmed M. Lanzer K. Randall T. D. Woodland D. L. Blackman M. A. (2008). Age-associated decline in T cell repertoire diversity leads to holes in the repertoire and impaired immunity to influenza virus. *J. Exp. Med.* 205 711–723 18332179 10.1084/jem.20071140 PMC2275391

[B110] Yunis E. J. Fernandes G. Smith J. Stutman O. Good R. A. (1973). Involution of the thymus dependent lymphoid system. *Adv. Exp. Med. Biol.* 29 301–306 4152933 10.1007/978-1-4615-9017-0_44

